# Epithelioid Hemangioendothelioma as a Model of YAP/TAZ-Driven Cancer: Insights from a Rare Fusion Sarcoma

**DOI:** 10.3390/cancers10070229

**Published:** 2018-07-10

**Authors:** John M. Lamar, Vijeyaluxmy Motilal Nehru, Guy Weinberg

**Affiliations:** 1 Department of Molecular and Cellular Physiology, Albany Medical College, Albany, NY 12208, USA; lamarj@mail.amc.edu; 2 Department of Oncology, University of Illinois College of Medicine, Chicago, IL 60612, USA; vmotil2@uic.edu; 3 Department of Anesthesiology, University of Illinois College of Medicine, and Jesse Brown VA Medical Center, Chicago, IL 60612, USA

**Keywords:** TAZ, epithelioid hemangioendothelioma, fusion sarcoma, transcriptional coactivators, Hippo signaling, YAP

## Abstract

Epithelioid hemangioendothelioma (EHE) is a rare soft-tissue sarcoma involving cells with histologic markers that suggest an endothelial origin. Around 90% of EHEs are caused by the fusion of Transcriptional Co-activator with a PDZ-motif (TAZ) with Calmodulin Binding Transcription Activator 1 (CAMTA1), a central nervous system-specific transcription activator. The 10% of EHEs that lack the TAZ–CAMTA1 fusion instead have a fusion of Yes-associated Protein (YAP) and Transcription Factor E3 (TFE3) genes (YAP-TFE3). YAP and TAZ are well-defined downstream effectors in the Hippo pathway that promote cell growth when translocated to the nucleus. The TAZ–CAMTA1 fusion transcript is insensitive to the Hippo inhibitory signals that normally prevent this process and thus constitutively activates the TAZ transcriptome. In EHE, this causes tumors to form in a variety of organs and tissue types, most commonly the liver, lung, and bone. Its clinical course is unpredictable and highly variable. TAZ activation is known to contribute to key aspects of the cancer phenotype, including metastasis and fibrosis, and increased expression of TAZ is thought to be causally related to the progression of many cancers, including breast, lung, and liver. Therefore, understanding TAZ biology and the molecular mechanisms by which it promotes unregulated cell proliferation will yield insights and possibly improved treatments for both EHE as well as much more common cancers.

## 1. Introduction

Experiments of nature are spontaneous phenomena that offer insights into otherwise mechanistically opaque events. They are usually both rare and extreme occurrences, two features that provide a basis for innovation and understanding. Such events have much in common with the ‘Black Swan’ occurrences described by Nassim Taleb as unexpected outlier events that can have a dramatic impact on our world-view [1]. They can, importantly, lead to novel ideas and explanations for a variety of observable facts. This is found to be especially true in medicine generally and particularly in the relationship of medical genetics to biochemistry and molecular biology. Archibald Garrod’s interest in inborn errors of metabolism, for instance, began with the observation that urine in a baby’s diaper darkened on exposure to air. Curiosity about the underlying cause resulted in his identifying the first Mendelian genetic disorder in humans, alkaptonuria, and establishing its autosomal recessive inheritance. His landmark paper in 1902, “The incidence of Alkaptonuria: A study of Chemical Individuality”, effectively pioneered biochemical genetics as an entirely new medical discipline (see here [2]). The study of rare, inherited forms of cancer has similarly informed the molecular mechanisms underlying a wide variety of normal and pathologic cellular process regulating cell proliferation, survival, and death.

Epithelioid hemangioendothelioma (EHE), a rare, translocation-based, soft-tissue sarcoma, is one such experiment of nature that can provide meaningful insights into the connection of genotype to the clinical phenotypes of cancer. The underlying genetic alteration in most EHEs is a fusion of the WW Domain-containing Transcription Regulator Protein 1 gene (WWTR1) with Calmodulin Binding Transcription Activator 1 (CAMTA1 gene). In 2013, a second fusion event between the Yes-associated Protein (YAP) and Transcription Factor E3 (TFE3) genes (YAP-TFE3) that occurs in around 10% of all EHEs was identified [3], but this fusion has not been as widely studied. The WWTR1 gene encodes the Transcriptional Co-activator with a PDZ-motif (TAZ), and the fusion results in constitutive activation of the TAZ transcriptome, which, as detailed below, drives a transformed phenotype, resistance to anoikis, and increased colony formation in soft agar [4]. Resistance to anoikis, or cell death after detaching from the culture substrate, is a particularly relevant corollary to the clinical phenotype of EHE, which often presents as widely metastatic disease when patients are first diagnosed. Unregulated TAZ expression also contributes to metastasis in other, more common cancers [5,6,7,8]. Patients with EHE can survive many years despite having widespread disease. This runs counter to classical staging systems used in most cancers [9] where disease progression and clinical outcomes are closely linked. In short, widespread cancer generally means aggressive disease and is correlated with limited life expectancy. However, this is not the case in EHE where the disease can be both widespread and dormant for years. This unusual feature, and more specifically the uncoupling of metastasis from adverse clinical outcomes, suggests that EHE might serve as a model for the cancer phenotype related uniquely to TAZ activation since patients with disseminated disease can survive for long periods without the co-morbidities that confound outcome studies of most cancers. This particular correlation of molecular to clinical phenotypes makes studying EHE a valuable model for dissecting and understanding the complex biology of TAZ in common cancers and EHE alike independent of the generally adverse effects of metastasis. This offers the hope of identifying treatments that reduce metastasis, and other TAZ-specific aspects of the malignant phenotype, thereby improving the lives of patients with EHE and, potentially, other more common cancers.

## 2. The EHE Phenotype

Mallory first used the term hemangioendothelioma in 1908 to describe all endothelial proliferations [10]. The disease later identified as EHE was first described by Dail and Leibow as a broncho-alveolar carcinoma in 1975 [11]. The term “epithelioid hemangioendothelioma” (EHE) was first used by Weiss and Enzinger in 1982 [12] to delineate an uncommon vascular tumor of soft tissue and bone with overlapping benign and malignant features that was often misidentified as a carcinoma. EHE accounts for less than 1% of all vascular tumors and arises from endothelial or pre-endothelial lineage cells. Corrin and colleagues [13] used immunohistochemical staining to confirm the presence of tumor cells capable of differentiating along an endothelial cell lineage and this was later confirmed by Weldon-Linne et al. using electron microscopy to reveal factor VIII antigen associated malignant cells with diffuse cytoplasmic staining [14].

### 2.1. Clinical Features

EHE usually presents in adulthood (median age at diagnosis in several case series is generally ~40 years with reported ranges of ~8–80 years) with a (1.5:1) bias among females. Affected organs vary, with liver (21%), lung (12%), and bone (14%) being the most commonly reported sites, but involvement of the central nervous system, skin, and oral cavity have also been documented [15]. It is an unusual neoplasm due not only to its rarity but also because of its presentation and clinical course. About a quarter to one third of the patients are asymptomatic at diagnosis since many EHEs are first identified as incidental findings on radiographic studies. When symptoms are present, they are usually non-specific with pain, anorexia, and weight loss being most common. Rarely, hepatic EHE may present as hepatic vein thrombosis (i.e., Budd–Chiari syndrome), but this is also seen with other malignancies, such hepatocellular carcinoma and renal, adrenal, or gastric cancer, and is thus not a specific symptom of EHE. The clinical course of EHE ranges from indolent tumors that lay dormant for years to highly aggressive disease with tumors that cause substantial morbidity and mortality in a matter of months. No reliable prognostic features are known for this rare low to intermediate grade vascular neoplasm, but attempts have been made to elucidate factors that could affect the clinical outcomes. Deyrup et al. correlated tumor size (>3 cm) and histologic appearance (more than three mitotic figures per high-powered field) with reduced 5-year survival in EHE (59% versus 100%) [16]. This contradicted a large clinic-pathologic study by Makhlouf and Ishak that found that the histology of the tumor, including nuclear pleomorphism and mitotic count, were of little value in predicting clinical outcomes because mitotic counts were low in both low-grade and aggressive tumors [15]. High cellularity, progressive disease-like ascites, and pleural effusion (lesions without distinct borders) portended poorer outcome [16]. Lau et al. [9] proposed a staging system where disease comprising entirely discrete tumors (Pattern A) correlated with longer survival than seen in non-discrete disease (Pattern B, including ascites, pleural effusion, etc.); patients converting from Pattern A to B had intermediate survival. The natural history of EHE is unpredictable, with a median overall survival of 75 months in a study comparing vascular hepatic neoplasms [17]. Even patients with metastatic disease at onset of diagnosis had overall survival that was superior to other malignancies of similar characteristics. About 15% of patients with EHE have recurrence after treatment and 20–30% had regional metastasis [18].

### 2.2. Diagnosis

Imaging, histology, and immunohistochemistry IHC are helpful in managing EHE, but are not always sufficient to accurately diagnose this rare vascular tumor. Therefore, diagnosing EHE can be a challenge and due to its rarity and variable clinical presentation it is often initially misdiagnosed. Laboratory abnormalities in blood counts are infrequently present and are rarely specific. Imaging is an essential tool in the detection of EHE with computed tomography (CT) and magnetic resonance imaging (MRI) being the most commonly used modalities. Some specific patterns on CT in patients with pulmonary epithelioid EHE include multiple pulmonary nodules, diffuse infiltrative pleural thickening, and multiple reticulo-nodular opacities. Similarly, some recurring patterns on CT/MRI in patients with hepatic EHE include multiple nodular lesions with calcifications and peripheral enhancement (lollipop sign).

Histology and immunohistochemistry were key to diagnosing EHE before the responsible fusions were identified. Tumor cells typically show cords or nests of epithelioid endothelial cells in myxohyaline stroma; about 50% of tumors are associated with or seen as an outgrowth of a pre-existing vessel. The cell nucleus is typically bland although 25% of tumors exhibit some cytological atypia. Immunohistochemical confirmation of endothelial differentiation can be helpful in confirming the diagnosis, and EHE cells typically stain strongly positive for CD31/platelet endothelial cell adhesion molecule (PECAM-1), CD34 (cell surface glycoprotein), or erythroblast transformation-specific related gene (ERG) [19]. The clinical and histological differential diagnosis of EHE typically includes hemangioma, hemangiosarcoma, myoepithelial tumors, and carcinoma. Both hemangioma and angiosarcoma express CD31 and CD34, and angiosarcoma also expresses cytokeratin, making the diagnosis of these tumors potentially challenging. However, identification of the WWTR1–CAMTA1 and YAP–TFE3 fusions is diagnostic and gives clinicians a potentially targetable therapeutic marker. Fluorescence in situ hybridization and quantitative polymerase chain reaction (qPCR) can rapidly identify the WWTRI–CAMTA1 fusion gene. However, in a small number of patients lacking this fusion, the presence of YAP–TFE3 may be identified by the presence of diffuse nuclear immunohistochemical staining of TFE3 [20,21]. This subset also displays distinct histologic features from conventional EHE, such as the presence of focal vasoformative areas and tumor cells with voluminous eosinophilic cytoplasm [20].

## 3. Molecular Pathology

### 3.1. The WWTR1–CAMTA1 Fusion and Its Role in EHE

The first clue about genetic drivers of EHE came in a study in 2001 that identified a chromosomal translocation involving chromosomes 1 and 3 (t(1;3)(p36.3;q25)) in EHE biopies [22]. Two subsequent studies in 2011 described this translocation as a fusion between WWTR1 and CAMTA1 [23,24]. Both of these studies examined numerous vascular sarcoma biopsies and found that the translocation was present in nearly all EHE samples, but not in other vascular tumors. Roughly 90% of EHE samples contain WWTR1–CAMATA1 fusions [3,19,25,26]. The remaining 10% typically contain the YAP–TFE3 fusion [3].

The WWTR1–CAMATA1 gene fusion joins the amino terminus of WWTR1 with the carboxyl terminus of CAMTA1 and is under the transcriptional control of the WWTR1 promoter [24]. TAZ, the product of the WWTR1 gene, is a downstream effector of the Hippo pathway that, when inappropriately active, can drive the development and progression of numerous cancers [5,6,7,8]. TAZ normally has important roles in early embryonic development, organogenesis, organ size determination, and tissue repair [8,27,28,29]. The CAMTA1 gene encodes a transcription factor that has been implicated as a candidate tumor suppressor in neural cancers [30,31,32,33]. This gene is found in essentially all multicellular organisms, but its expression in humans is limited largely to the brain, and little is known about the function of CAMTA1 in humans. Studies suggest it normally plays a role in memory, behavior, and neuronal function [34,35,36,37,38] as well as visual response to light [39] and myocardial linage commitment [40]. Paradoxically, in different contexts CAMTA1 is a candidate tumor suppressor in neuroblastoma [41] and glioma [30].

**Figure 1:**
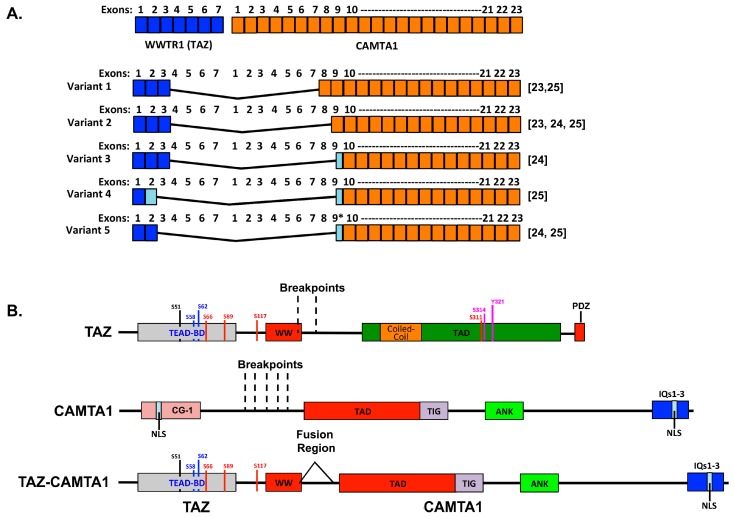
Schematic of the WWTR1–CAMTA1 fusions and their protein structures. (**A**) Shown is the exonic structure of the WWTR1 and CAMTA1 transcripts (top) as well as the fusion transcripts identified in the literature to date [23,24,25]. Light blue boxes indicate partial exons. (**B**) Protein structures for TAZ, CAMTA1, and the fusion with key domains indicated. TEAD-BD, TEAD binding domain; WW, WW domain; TAD, transactivation domain; PDZ, PDZ binding motif; CG-1, CG-1 DNA binding domain; NLS, nuclear localization signal; TIG, transcription factor immunoglobulin domain; ANK, ankyrin repeats; IQ, IQ calmodulin-binding motifs. Regulatory phosphorylation sites on TAZ are also shown: S66, S89, S117, and S311 are LATS phosphorylation sites; S58 and S62 are glycogen synthase kinase 3 beta phosphorylation sites; S51 is critical for TEAD binding; S314 is a CK1ε/δ kinases phosphorylation site; and Y321 is a Src family kinase/Abl kinase phosphorylation site.

Several variations of the WWTR1–CAMATA1 fusion with slightly different breakpoints have been identified (Figure 1) [23,24,25]. All variants identified to date contain the TEAD binding domain, 14-3-3 binding motif, and all or most of the WW domain of TAZ fused to the CAMTA1 transactivation domain (TAD), TIG domain, ankyrin repeats, and IQ domains [23,24,25]. Tanas and colleagues were the first to characterize the tumorigenic mechanism of action of the WWTR1–CAMATA1 gene fusion [4]. They generated a construct encoding the fusion protein and compared its function to that of full-length TAZ, full-length CAMTA1, and to the truncated forms of TAZ and CAMTA1 that are contained in the fusion. Expression of the fusion protein promoted cellular transformation and adhesion-independent growth in NIH3T3 cells, whereas truncated or full-length TAZ or CAMTA1 did not. They also used transcriptional profiling to compare genes regulated by the fusion to those regulated by full-length TAZ and full-length CAMTA1. This revealed that there was significant overlap between the transcriptional changes induced by the fusion and TAZ, but not between the fusion and CAMTA1. This suggests that the fusion can drive a TAZ/TEAD (TEA Domain family member)-like transcriptional program. Consistently, the fusion activates a TEAD-responsive transcriptional reporter construct [4]. Furthermore, the oncogenic properties of the fusion require TEAD binding since S51A mutation, which prevents TEADs from binding TAZ [42], prevented the fusion from promoting cellular transformation [4]. Large Tumor Suppressor Homolog (LATS)-mediated phosphorylation of TAZ on serine 89 is known to repress TAZ transcriptional activity by promoting 14-3-3 binding and cytoplasmic sequestration [43]. Serine 89 is still phosphorylated by LATS in the fusion protein [4], and the phosphorylated fusion still binds 14-3-3 (personal communication Brian Rubin, 2018). However, the fusion is not sequestered in the cytoplasm in response to phosphorylation [4]. The CAMTA1 portion of the fusion also influences its oncogenic activity because it contributes not only the transactivation domain, but also a non-canonical nuclear localization signal (NLS) that is essential to drive the fusion into the nucleus [4]. Thus, it appears this NLS may be sufficient to promote the translocation of the fusion even if it is phosphorylated and bound by 14-3-3. Collectively, these studies suggest that EHE is driven by inappropriate TAZ-TEAD transcription resulting from the WWTR1–CAMATA1 gene fusion.

Inappropriate TAZ transcriptional activation and/or Hippo pathway dysregulation drives tumor development, progression, and metastasis in many cancer types [5,6,7,8]. This includes other sarcomas, and several studies have found that loss of Hippo pathway activity or forced activation of YAP or TAZ can promote sarcoma formation and progression (reviewed in [44,45]). Indeed, aberrant YAP/TAZ activity can promote the formation and progression of osteosarcoma [46,47], rhabdomyosarcoma [48,49,50,51,52,53], and soft-tissue sarcomas [54]. In addition, evidence suggests that many of the most common sarcoma types have genetic alterations in the Hippo pathway [55] or increased YAP or TAZ protein expression [56]. While these studies suggest that mesenchymal cells are particularly vulnerable to deregulation of the Hippo-YAP/TAZ pathway, it is important to note that the WWTR1–CAMATA1 fusion is unique to EHE, and that other genetic alterations in the Hippo pathway often found in other cancers are not found in EHE. This suggests that there is something unique about EHE relative to other cancers.

Understanding why EHE and the WWTR1–CAMATA1 fusion share this unique connection is an important step in understanding and curing this disease. If the only consequence of the fusion is constitutive TAZ-mediated transcription, then TAZ activation should be sufficient to promote EHE and the fusion should occur in other cancers where TAZ activation promotes tumorigenesis. The fact that this is not the case suggests that the CAMTA1 portion of the protein provides additional function beyond just recruiting the TAZ portion to the nucleus and driving transcription. Consistently, while there was significant overlap between the genes regulated by the fusion and TAZ in NIH3T3 cells, there were many genes that were unique to each. These differences in gene expression may account for some of the specificity of the fusion to EHE. Alternatively, it may be that the EHE cell of origin, which is currently unknown, is particularly susceptible to the fusion but not other alterations in the Hippo-YAP/TAZ pathway. Identification of the cell of origin is likely a critical step in understanding how the fusion drives disease formation and progression, and would also aid in the generation of EHE model systems, the lack of which hinders research efforts.

### 3.2. Overlap among Clinical Features of EHE and TAZ-Driven Cell Phenotypes

Since the WWTR1–CAMATA1 fusion that drives EHE results in activation of a TAZ-like transcriptional program, it is not surprising that many clinical features of EHEs are also associated with TAZ activation in other cancer types and/or pathological processes. For example, in many diseased tissues TAZ activation plays important roles in driving fibrosis and can promote the differentiation of fibroblasts into highly contractile myofibroblasts [57,58,59,60,61,62,63,64,65]. YAP/TAZ activation is also important for the pro-oncogenic functions of cancer-associated fibroblasts [66,67,68]. Connective Tissue Growth Factor (CTGF), a known YAP/TAZ target gene, has established roles in fibrosis and cancer progression [69,70,71], and many other known YAP/TAZ target genes also contribute to a fibrotic or activated stroma. Consistently, EHE tumors are typically described as having a prominent stroma with abundant fibrous connective tissue [16,26]. A high percentage of EHE patients present with metastatic disease [9] consistent with the lack of anoikis seen in cells transformed with the WWTR1–CAMATA1 gene fusion [24]. TAZ is also an important driver of metastasis in a variety of other cancer types (reviewed in [5,6]).

## 4. Treatment

The extreme infrequency and highly variable rate of progression of EHE currently preclude establishing well-defined study designs and therefore guidelines for its treatment. Moreover, the unpredictable natural history of EHE makes the confident interpretation of clinical response to any medical therapy very challenging. For instance, it is difficult to ascribe benefit to a specific treatment in a disease that normally advances and remits intermittently with no clear pattern. The potential heuristic benefit of any trial is weak in a disease so rare that adequate powering to establish efficacy is nearly impossible. These factors also help explain the tendency for many physicians to choose a conservative, wait-and-watch approach to patients with indolent disease, using CT or MRI imaging at regular intervals to surveille disease progression. This also underscores the importance of an as yet unmet need for a robust biomarker of EHE activity to allow intervention at an early indication of progression.

Despite the inherent limitations to medical progress in a very rare disease, a few decades of experience have taught us some things about treating EHE. Surgical excision of solitary lesions, especially those in the extremities, can be curative. Unfortunately, most patients present with systemic (metastatic) disease. In this case, the approach depends on the organ(s) involved and the accessibility of lesions to surgery or interventional procedures. For instance, multifocal liver disease, one of the most common presentations of EHE, is routinely treated with interventional radiologic procedures, including radiofrequency ablation, irreversible electroporation, or embolization. Several large-scale clinical series have also shown success with liver transplantation in EHE [72,73]. These studies have shown that metastatic disease does not adversely impact outcome and is therefore not a contraindication to transplantation. Interestingly, survival of EHE patients after liver transplant mirrors overall liver transplant survival curves [74,75,76]; this lack of additive mortality suggests that liver transplant itself might confer a specific benefit to EHE-related outcome per se.

### 4.1. Orthodox Approaches to Treatment

Current approaches to metastatic EHE include various conventional cytotoxic and anti-angiogenic chemotherapy regimens, alone or in combination [77,78,79,80]. An early meta-analysis of treatment outcomes by Mehrabi et al. showed the 1, 3, and 5-year survival rates irrespective of treatment modality to be 83.4%, 55.7%, and 41.1%, respectively [16]. As there are no prospective trials to give us a more evidence-based treatment algorithm, case reports of therapeutic interventions play an important role in informing the management of EHE. The cytotoxic parent agents (as well as their congeners) used most commonly in clinical series are carboplatin, paclitaxel, and adriamycin; other reports include etoposide, ifosfamide, dacarbazine, cyclophosphamide, and vincristine [78,81,82].

Targeted therapy also shows promise in treating EHE. The PALETTE study was the first randomized phase III trial that showed efficacy of pazopanib in the treatment of sarcoma. Subsequently, a few case reports have documented therapeutic success of targeted agents. A case report evaluating pazopanib in metastatic pulmonary EHE, for instance, demonstrated stable disease over 24 months [83], while another report demonstrated long-term partial response in a patient with multinodular liver EHE on sorafenib [84]. Although prospective research for newer agents to treat EHE is limited by its rarity, there are a few ongoing clinical trials addressing this deficiency. Phase II trials evaluating MEK inhibitor trametinib and anti-microtubular agent eribulin as potential therapeutic agents for EHE are currently underway.

Use of anti-angiogenic agents is rational given that EHE is a disease of unregulated proliferation in a cell with endothelial markers [85,86]. Drugs used in EHE have included thalidomide, bevacizumab (vascular endothelial growth factor inhibitor), and apatinib (specific VEGFR2 inhibitor) [87,88,89]. Case reports combining the use of cytotoxic chemotherapy regimens, such as carboplatin or pemetrexed with the addition of bevacizumab, to treat pulmonary EHE also show promise [90]. One case report in a patient with metastatic EHE treated with thalidomide after failure of interferon documents radiological and clinical stable disease for 7 years [88]. Conversely, there are also case reports that document progression or partial response on thalidomide [87,89]. The mTOR inhibitor sirolimus has shown some promise in advanced EHE. A recent case series of 18 patients with advanced and progressing EHE treated with sirolimus showed clinical benefit with 56% of patients surviving >24 months [91]. An interesting rationale for using mTOR inhibitors in EHE is found in the report of Hansen et al. [92] showing that YAP/TAZ induces expression of the high affinity leucine transporter (LAT1), which increases the uptake of leucine, an activator of mTORC1 that can lead to cellular proliferation.

### 4.2. Untested Potential Treatments

Less-conventional approaches with some theoretical rationale that have yet to be used clinically include verteporfin, a porphyrin derivative approved for phototherapy of macular degeneration, which is thought to inhibit interaction of YAP, and presumably TAZ, with TEAD, thereby impairing transcription of downstream signals [50,93,94,95,96,97]. Tanas et al. [4] showed that expression of the TAZ transcriptome in a transformed cell model of WWTR1/CAMTA1 fusion appears to be independent of upstream signals. However, if TAZ expression is shown in humans with EHE to be sensitive to upstream inputs, then a variety of additional potential therapies might come into play. For instance, inhibition of mevalonate synthesis (e.g., with statins) could potentially reduce fusion expression since mevalonate stimulates YAP/TAZ activity [98,99]. YAP/TAZ are downstream effectors of the Hippo pathway, which responds to extracellular matrix stiffness among other cues to regulate cell proliferation. Therefore, drugs such as losartan that ‘soften’ the matrix could possibly inhibit YAP/TAZ activity to the extent that the fusions proteins are sensitive to upstream inputs and modulation. A variety of similar potential treatments could be identified on the basis of their interaction with Hippo and other upstream signals as well as inhibition of YAP/TAZ-TEAD interaction, TEAD activation, or downstream effectors of TEAD. Biologicals could also be useful in targeting or possibly treating EHE. For instance, TRC105, a monoclonal antibody to endoglin, an endothelial-cell-specific surface marker, is currently undergoing clinical trials in recurrent gliobastoma, advanced hepatocellular carcinoma, and metastatic renal cell carcinoma [100,101]. Endoglin is highly expressed in EHE cells [102] suggesting that TRC105 could be useful in EHE. Eventually, large throughput screens of small molecule libraries will be another useful strategy for identifying candidate treatments when meaningful models of EHE are established in a cell culture system, patient derived xenograft, or genetically engineered mouse. It is also rational to consider immunotherapy in EHE. Interestingly, recent work reported that both YAP and TAZ promote PD-L1 expression in multiple cancer types, including melanoma, lung adenocarcinoma, non-small cell lung cancer, and breast cancer [103,104,105,106]. This increased PD-L1 expression was sufficient to inhibit T-cell function, suggesting that checkpoint inhibition could be of value. Another approach could include immunization against neo-antigens formed by the fusion protein and its products.

### 4.3. Strategies to Target the WWTR1–CAMTA1 Fusion to Treat EHE

An exciting future prospect for treatment is to specifically target and inhibit function of the disease-identifying fusion genes of WWTR1–CAMTA1 and YAP–TFE3. This would theoretically produce more durable outcomes than any standard or proposed medical regimens. Currently, the most promising approach is to target fusion–TEAD interaction. Given growing evidence showing that TAZ and YAP are important drivers of cancer development and progression, there is great enthusiasm for targeting YAP/TAZ–TEAD interaction generally, and this could potentially be very effective against EHE specifically. Although the fusion may be insensitive to Hippo pathway regulation, the list of Hippo-pathway-independent mechanisms of YAP/TAZ regulation continues to grow and several negative regulators of YAP and TAZ bind the WW domains [29,92,107], which are intact in the fusions described in EHE. Thus, it may also be possible to inhibit the fusion by activating these endogenous negative regulators. However, this will require a more thorough testing of which known TAZ regulators can also regulate the fusion protein. It may also be possible to directly regulate transcription of the fusion in a variety of cell types. Moreover, TAZ protein stability is also regulated by an N-terminal phosphodegron motif, which when phosphorylated by Glycogen synthase kinase-3 (GSK-3) leads to proteasomal degradation (Figure 1) [108]. This study further found that Phosphatidylinositol 4,5-bisphosphate 3-kinase (PI3K)/Protein kinase B (AKT) signaling represses GSK-3 leading to increased TAZ stability and function. The fusion protein also contains this phosphodegron motif, so it is reasonable to predict that the stability of the fusion would be regulated in a similar manner.

One potential problem with this strategy is that TAZ and YAP play important roles in organ development as well as normal cell function and repair in adult tissues. Systemic YAP/TAZ or TEAD inhibition may therefore result in as yet unpredictable adverse side effects. Another potential approach is to attempt to target the CAMTA1 portion of the fusion. Indeed, as mentioned above, the CAMTA1 portion of the fusion is required for its oncogenic activity because it contributes the transactivation domain and NLS that are essential for function [4]. Very little is known about the regulation of the CAMTA1 protein, and it would be interesting to explore whether post-translational modifications in the C-terminus of CAMTA1 influence its localization, stability, or transcriptional activity. miRNAs that target the 3′UTR of the CAMTA1 transcript or the coding region contained in the fusion might also regulate expression of the fusion. Though delivering miRNAs or silencing RNAs in general is not yet a feasible therapeutic approach in most tissues, it may be possible to identify targetable pathways that regulate these miRNAs.

A third approach may be to target the genes regulated by the fusion that are responsible for EHE development and progression. Numerous TAZ target genes are known, and many have established roles in cancer [5]. A more thorough characterization of the transcriptional changes mediated by the TAZ/CAMTA1 fusion protein may reveal potential targets. Tanas and colleagues [4] performed RNA sequencing on NIH3T3 cells expressing the fusion, but there is considerable variation in the genes regulated by TAZ across different cell types, and many known TAZ target genes are also context dependent. This means that the genes regulated by the fusion in fibroblasts may not be the same as the genes regulated in EHE cells. It will be important to identify TAZ/CAMTA1 target genes specifically in EHE samples or cells derived from EHE, and then confirm that they contribute to EHE development or progression. Transcriptional profiling of EHE cells in which the fusion has been targeted or inhibited would be the best way to identify fusion-regulated genes. However, EHE cell lines do not exist and have proven extremely difficult to generate. Clearly, a reliable genetically engineered mouse model of EHE would facilitate the identification of target genes as well as provide a system to test novel small molecule, biological, gene silencing/editing, or other putative therapies.

It is worth noting that inhibitors of the tankyrase class of PARP enzymes represent a potentially promising method of reducing YAP/TAZ activity in EHE. Zhao et al. showed that members of the angiomotin-like family of angiostatin binding proteins associate closely with YAP/TAZ and function effectively within the Hippo pathway to enhance their phosphorylation, thereby increasing cytoplasmic localization and degradation [109]. Conversely, knock down of AMOTL2 in canine kidney cells resulted in increased YAP/TAZ nuclear localization. Tankyrases associate with angiomotins and promote their degradation. Wang et al. found that tankyrase inhibitors functionally stabilized angiomotin proteins by reducing their rate of degradation, thereby also indirectly reducing nuclear localization of YAP/TAZ [110]. They further reported that tankyrase inhibitors reduced the growth rate of YAP-transformed cultured cells. Building on this finding, Troilo et al. showed that tankyrase inhibition can inhibit TEAD-dependent transcription [111]. In screening for small molecule inhibitors of TEAD activity, they identified a known tankyrase inhibitor. This molecule also eliminated YAP-dependent anchorage-independent cell growth in culture. They found that the inhibition of TEAD activity depended entirely on inhibition of angiomotin degradation. The biological importance of these relationships was further supported by studies showing that tankyrase inhibition can impair cell growth in lung cancer [112] and liver cancer [113] by reducing YAP activity. The potential for using tankyrase inhibitors in EHE depends in part on the degree to which the YAP/TAZ binding of angiomotin family proteins occurs in the fusion protein. This binding normally occurs at the WW domain, which is at least partly intact in most EHE fusions; future studies in genetically engineered mouse models or possibly immunoprecipitation with synthetic oligomers will help predict which patients’ fusions might be susceptible to treatment by tankyrase inhibition.

Finally, hypermethylation of Hippo-related promoters, such as LATS1 and LATS2 and MST1 and MST2, can contribute to tumor growth and aggressiveness in many cancers [114], including sarcomas [115]. This phenomenon has not been sufficiently studied in EHE yet, but future research will certainly address the contribution of epigenetics to fusion expression and clinical outcomes in patients with EHE, hopefully with the potential for opening additional lines of treatment.

## 5. Conclusions

Study of the molecular biology of EHE will lead to a better understanding of mechanisms specific to YAP/TAZ-induced cell proliferation and, eventually, improved treatment of both this rare disease and many of the more common cancers. Chief among the remaining hurdles are: establishing robust model systems (cell lines and transgenic animals); achieving a fine-grained dissection of the fusion’s function and regulation; and comprehensive elaboration of downstream targets. Genetic approaches hold great promise for treatment of EHE, a monogenic disorder. This includes, for instance, use of silencing RNAs specific to both the fusion transcript and its downstream effectors. Gene-editing strategies could also theoretically knock down fusion gene expression or potentially delete the underlying fusion from tumor cells altogether. Such advances could lead to potential therapies in a wide array of YAP/TAZ-driven cancers and thereby help many patients. Clearly, experiments of nature, no matter how rare, can reveal secrets with wide applicability.

## Abbreviations

AKT	Protein kinase B
CAMTA	Calmodulin Binding Transcription Activator 1
CT	Computed Tomography
CTGF	Connective Tissue Growth Factor
EHE	Epithelioid Hemangioendothelioma
ERG	ETS related gene
GSK-3	Glycogen synthase kinase-3
LATS	Large Tumor Suppressor Homolog
MRI	Magnetic resonance imaging
NLS	nuclear localization signal
PECAM 1	Platelet endothelial cell adhesion molecule 1
PI3K	Phosphatidylinositol 4,5-bisphosphate 3-kinase
qPCR	quantitative polymerase chain reaction
TAD	Transactivation Domain
TAZ	Transcriptional Co-activator with PDZ-binding Motif
TEAD	TEA Domain Family Member
TFE3	Transcription factor E3
UTR	untranslated region
WWTR1	WW Domain Containing Transcription Regulator 1
YAP	Yes Associated Protein
